# Microglia-targeting nanotherapeutics for neurodegenerative diseases

**DOI:** 10.1063/5.0013178

**Published:** 2020-09-08

**Authors:** Nanxia Zhao, Nicola L. Francis, Hannah R. Calvelli, Prabhas V. Moghe

**Affiliations:** 1 Department of Chemical and Biochemical Engineering, 98 Brett Rd., Rutgers University, Piscataway, New Jersey 08854, USA; 2 Department of Biomedical Engineering, 599 Taylor Rd., Rutgers University, Piscataway, New Jersey 08854, USA; 3 Department of Molecular Biology and Biochemistry, 604 Allison Rd., Rutgers University, Piscataway, New Jersey 08854, USA

## Abstract

Advances in nanotechnology have enabled the design of nanotherapeutic platforms that could address the challenges of targeted delivery of active therapeutic agents to the central nervous system (CNS). While the majority of previous research studies on CNS nanotherapeutics have focused on neurons and endothelial cells, the predominant resident immune cells of the CNS, microglia, are also emerging as a promising cellular target for neurodegeneration considering their prominent role in neuroinflammation. Under normal physiological conditions, microglia protect neurons by removing pathological agents. However, long-term exposure of microglia to stimulants will cause sustained activation and lead to neuronal damage due to the release of pro-inflammatory agents, resulting in neuroinflammation and neurodegeneration. This Perspective highlights criteria to be considered when designing microglia-targeting nanotherapeutics for the treatment of neurodegenerative disorders. These criteria include conjugating specific microglial receptor-targeting ligands or peptides to the nanoparticle surface to achieve targeted delivery, leveraging microglial phagocytic properties, and utilizing biocompatible and biodegradable nanomaterials with low immune reactivity and neurotoxicity. In addition, certain therapeutic agents for the controlled inhibition of toxic protein aggregation and for modulation of microglial activation pathways can also be incorporated within the nanoparticle structure without compromising stability. Overall, considering the multifaceted disease mechanisms of neurodegeneration, microglia-targeted nanodrugs and nanotherapeutic particles may have the potential to resolve multiple pathological determinants of the disease and to guide a shift in the microglial phenotype spectrum toward a more neuroprotective state.

NOMENCLATURE

AD: Alzheimer's disease
AJs: adherens junctions
ALS: amyotrophic lateral sclerosis
AM: amphiphilic macromolecules
ApoE: apolipoprotein E
ApoE3-rHDL: apolipoprotein E3-reconstituted high density lipoprotein
ASYN: alpha synuclein
Aβ: amyloid beta
BBB: blood-brain barrier
CNS: central nervous system
CSF: cerebrospinal fluid
CTE: chronic traumatic encephalopathy
CZ NPs: ceria-zirconia nanoparticles
DA: dopaminergic
DAM: disease-associated microglia
DAMP: damage-associated molecular patterns
DLB: dementia with Lewy bodies
EGCG: epigallocatechin gallate
FDA: Food and Drug Administration
FTD: frontotemporal dementia
HIV-1: human immunodeficiency virus type-1
IN: intranasal
iNOS: inducible nitric oxide synthase
LincRNA-Cox2: long intergenic non-coding RNA-cyclooxygenase-2
LPS: lipopolysaccharide
MS: multiple sclerosis
MSC: mesenchymal stem cell
NAMP: neurodegeneration-associated molecular patterns
NO: nitric oxide
NPs: nanoparticles
NSAIDS: non-steroidal anti-inflammatory drugs
PAMP: pathogen-associated molecular patterns
PCL: poly-ε-caprolactone
PD: Parkinson's disease
PDD: Parkinson's disease dementia
PEG: polyethylene glycol
PEI: polyethylenimine
PGE2: prostaglandin E2
PHOX: phagocyte oxidase
PMMA: polymethylmethacrylate
PRRs: pattern recognition receptors
RAGE: receptor for advanced glycation end products
RIPK1: receptor-interacting serine/threonine-protein kinase 1
ROS: reactive oxygen species
SOD1: superoxide dismutase
TAT: transactivator of transcription
TfR: transferrin receptor
TJs: tight junctions
TLR: toll-like receptor
TREM2: triggering receptor expressed on myeloid cells 2

## THE ROLE OF MICROGLIA IN NEURODEGENERATIVE DISEASES

### *Microglial functions in healthy* vs *diseased brain*

Microglia are the resident immune cells of the brain and are derived from primitive myeloid progenitors that arise during embryonic development.^1^ They represent 5%–12% of cells in the healthy CNS, with different brain regions possessing different microglial subpopulations.^2^ Microglia serve as the initial mode of defense by generating both innate and adaptive immune responses upon disturbance to homeostasis. In the healthy brain, microglia possess a surveillance phenotype, consisting of a ramified morphology with long cytoplasmic protrusions that allow them to survey their environment from a resting state.^3^ By continuously monitoring changes in the brain, microglia eliminate pathogens and preserve the health of different cell types of the CNS.^4^ Microglia display various signaling immunoreceptors to interact with extracellular species, including the TLR2 and TLR4 toll-like receptors, CR3 and CR4 phagocytic receptors, and CD36 and CD204 scavenger receptors.^2^ Because of their high degree of phenotypic and functional plasticity, microglia exhibit robust responses to changes in their microenvironment.^5^ These responses can be neurotoxic or neuroprotective because microglia are involved in both physiological and pathological conditions, protecting the CNS in physiological conditions and enhancing disease progression in pathological conditions. Their resting state morphology also allows them to physically interact with the synapses of neurons to regulate neuronal activity.^6^ They play important roles in mediating neuronal activity by preserving the neural environment, responding to injury, and facilitating repair.^3^ Specifically, microglia help promote neurogenesis, reshape neuronal circuitry, mediate neuronal transmission, and regulate synaptic pruning and apoptosis.^2,4^ Overall, there is a high degree of crosstalk between microglia and neurons under normal physiological conditions.

When homeostasis is compromised, microglia respond to changes in their microenvironment by transforming into a reactive phenotype marked by an ameboid morphology and the contraction of processes.^2^ Microglia in a resting state display a low-level expression of genes that contribute to the CNS inflammatory response, and this response is further dampened by neurotrophic factors released by neurons.^7^ Upon detection of injury or damage, microglial activation results in the upregulation of many cell surface receptors, the release of various complement factors, and changes to the cytokine profile.^2^ This activation is a plastic and dynamic process that has been shown to be brain region-specific.^6^ Microglial activation is often an early and sustained response in neurodegenerative diseases, leading to oxidative stress and neuroinflammation as a result of systemic inflammation. Microglial activation states have historically been described in terms of an “M1” pro-inflammatory phenotype and an “M2” anti-inflammatory phenotype although this distinction oversimplifies the dynamic range of phenotypes that microglia can possess.

**FIG. 1.**
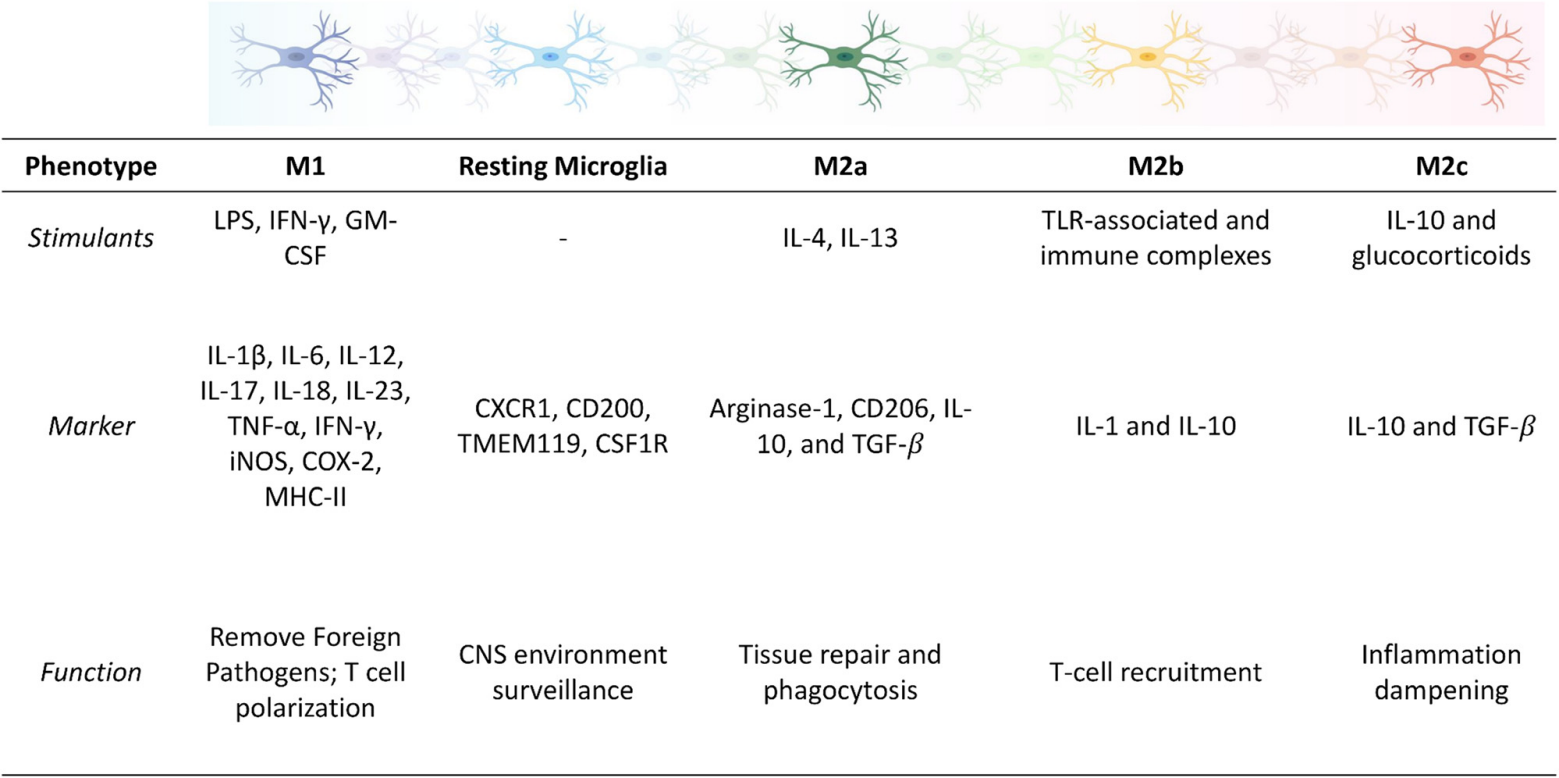
Spectrum of microglial phenotypes upon stimulation. Depending on the type of stimulant, the microglial phenotype can be roughly categorized into pro-inflammatory (M1) or anti-inflammatory (M2×).^2–4,7^ While simply categorizing microglia into only two main categories does not do justice to their diverse function in the CNS hemostasis, the corresponding phenotypic markers listed here are only meant to guide the characterization of microglia but not to restrict the interpretation of diverse functional roles that different microglial phenotypes play in the CNS.

The classically activated M1 phenotype is associated with disruptions in homeostasis and pro-killing functions, resulting in the release of pro-inflammatory cytokines as a first line of defense against infection or injury. The following phenotypic markers are involved in the M1 immune response: IL-1β, IL-6, IL-12, IL-17, IL-18, IL-23, TNF-α, IFN-γ, iNOS, COX-2, MHC-II, ROS, reactive nitrogen species, and prostaglandin E2 (PGE2).^4^ The upregulation of these markers is associated with increased oxidative stress, neuroinflammation, and ultimately exacerbated neurodegeneration. The alternatively activated M2 phenotype is associated with sustained homeostasis and inflammation dampening, resulting in the release of neurotrophic factors and anti-inflammatory cytokines to promote healing and tissue repair. Microglial M2 phenotypes can be further divided into the M2a, M2b, and M2c subtypes. The M2a subtype involves tissue repair and phagocytosis and is activated by IL-4 and IL-13, resulting in the upregulation of arginase-1, CD206, IL-10, and TGF-β.^3,4^ The M2b subtype involves T-cell recruitment and is activated by TLRs and immune complexes, resulting in the upregulation of IL-1 and IL-10. The M2c subtype is involved in inflammation dampening and healing and is activated by IL-10 and glucocorticoids, resulting in the upregulation of IL-10 and TGF-β.^3,4^ Due to the high degree of crosstalk between microglia and neurons, activation of a pro-inflammatory microglial phenotype can disrupt normal neuron-microglia communication, resulting in aberrant neuronal signaling, neuronal dysfunction, and neuronal loss that contributes to pathogenesis in neurodegenerative diseases.^2,7^ The phenotypes of microglia mentioned above are summarized and illustrated in Fig. 1.

Many research groups now define context-specific microglial activation and phenotype as a measure of the diversity of microglial functions.^8^ Single-cell RNA sequencing analysis has revealed the existence of a subset of microglia that display a unique transcriptional and functional signature in neurodegenerative conditions, termed disease-associated microglia (DAM).^9,10^ Induction of this DAM phenotype is initiated by the recognition of neurodegeneration-associated molecular patterns (NAMPs) by receptors expressed on microglia, triggering their transition into DAM.^9,10^ This phenotype is shared across various neurodegenerative diseases, such as Alzheimer's Disease (AD), Amyotrophic Lateral Sclerosis (ALS), and Multiple Sclerosis (MS), and in response to aging.^10,11^ Researchers have also distinguished multiple disease stage-specific cell states within DAM populations, which also suggests that these DAM phenotypes may occur along the transition from early stage (i.e., protective/beneficial functions) to late stage (neurotoxic) microglia.^12,13^

### Mechanisms of how microglia kill neurons

**FIG. 2.**
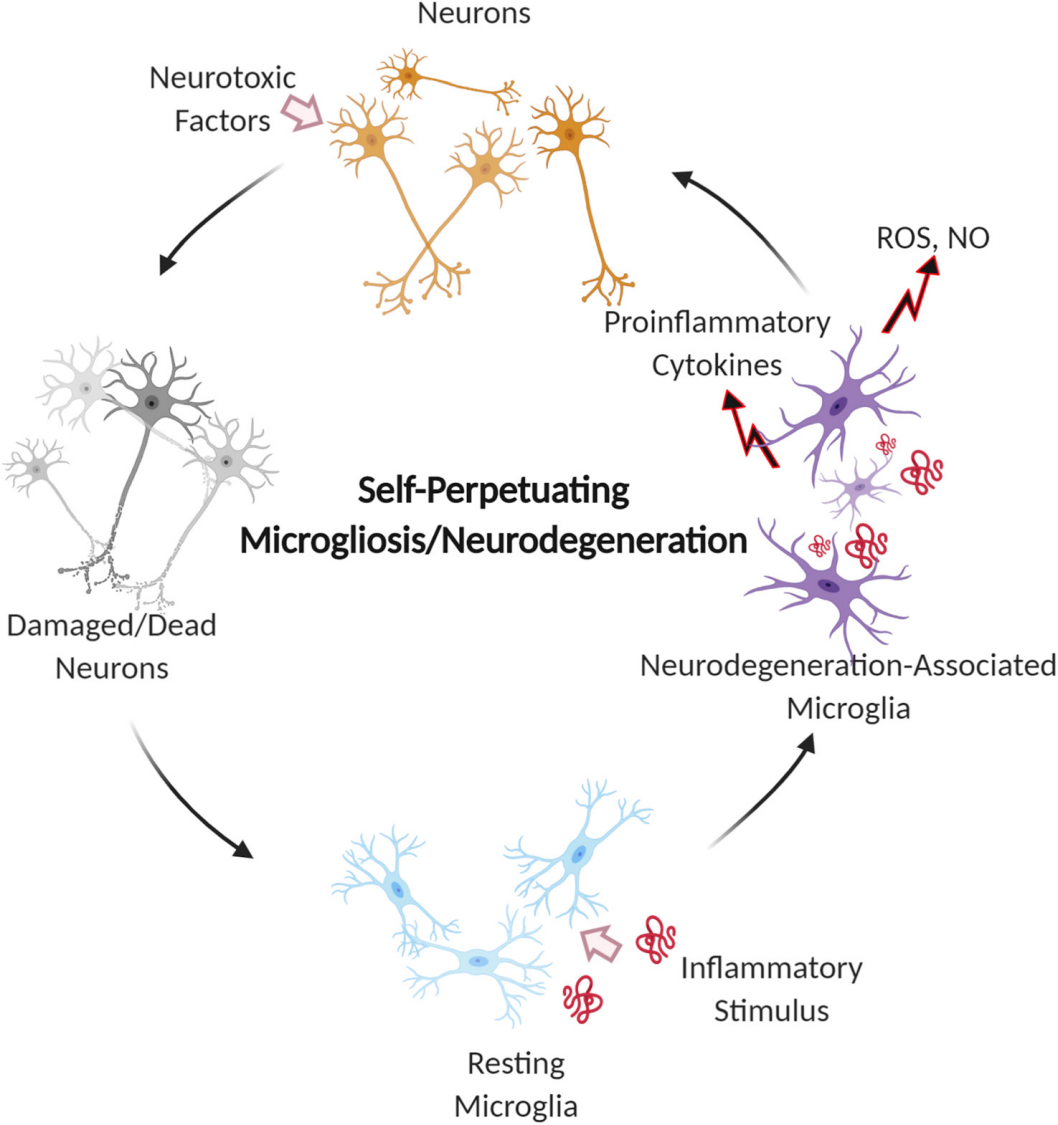
Self-perpetuating cycle of microglial activation and neuron damage during neurodegeneration. Microglia recognize, uptake, and phagocytose inflammatory stimuli, the prolonged exposure to which results in chronic microglial activation. The activation of microglia initiates the release of neurotoxic free radicals and pro-inflammatory cytokines, which, in turn, leads to neuronal damage and further stress on microglia, leading to microgliosis.

Sustained microglial activation, known as microgliosis, is believed to play a role in exacerbating neuronal loss in neurodegenerative diseases due to oxidative stress and neuroinflammation.^14,15^ Research has shown that activated microglia coincide with regions of neuronal cell death and phagocytose dying neurons.^6^ Microglia are part of a self-propelling cycle where microgliosis causes an inflammatory response that leads to neuronal death, and this neuronal death promotes further microgliosis^3^ (Fig. 2). Thus, microglia help amplify the progressive neurodegeneration in diseases by contributing to neuronal dysfunction. It is believed that although microglia are associated with neuronal loss, they are likely involved in the escalation of neuronal loss rather than the initial cause.^6^ In AD and Parkinson's Disease (PD), microglia have been shown to generate neurotoxic species following the internalization of amyloid-β (Aβ) and α-synuclein (ASYN), respectively, leading to neuronal damage.^2^ As neurodegenerative diseases progress, the communication between microglia and neurons is further disrupted, resulting in deregulation and abnormal activation that leads to greater neuronal loss.

There are several specific mechanisms by which activated microglia cause neuronal dysfunction and death. Inflammatory stimuli released by activated microglia increase the expression of phagocyte NADPH oxidase (PHOX) to produce superoxide and other reactive oxygen species (ROS) that stress or kill neurons.^16^ Increased PHOX expression also promotes microglial proliferation, leading to an increase in the production of ROS. Another mechanism involves the expression of inducible nitric oxide synthase (iNOS) in activated microglia, which results in the production of nitric oxide (NO) and other ROS. High levels of NO lead to neuronal death via inhibition of mitochondrial cytochrome oxidase in neurons, which results in glutamate release and excitotoxicity.^17^ Pro-inflammatory cytokines released by activated microglia, such as TNF-α and IL-1β, can induce apoptosis and result in neuronal death. Microglial phagocytosis of stressed neurons may also accelerate cell death. Reactive oxygen and nitrogen species produced by activated microglia contribute to neuronal stress and trigger exposure of phosphatidylserine on the surface of neurons, which signals for microglia to phagocytose the neurons.^18^

### Effects of aging on microglia

Neurodegenerative diseases such as AD and PD are age-related, and the prevalence of these diseases is expected to increase over time due to higher life expectancies. Microglia play a significant role in age-related changes. Aging is associated with an increase in oxidative stress, disrupting the normal protective role of microglia in preserving neural integrity.^7^ Aging also leads to microglial activation, which results in a shift in the cytokine profile toward the pro-inflammatory phenotype with decreased phagocytosis and increased production of ROS.^5^ Activated microglia are less effective at clearing neurotoxic protein aggregates such as Aβ and ASYN. Due to the chronic nature of aging, microglia eventually cease proliferation and become senescent, which drives the progression of age-related neurodegenerative diseases. Microglia are normally able to regenerate, but during aging, telomere shortening occurs, which results in cellular dystrophy and senescence.^19^ The morphological characteristics of senescent microglia include the elimination of processes, formation of spheroids, and fragmentation of the cytoplasm.^20^ Unlike highly proliferative cells, microglia have limited telomerase activity and, thus, limited replication potential. The degeneration of senescent microglia results in a loss of their neuroprotective functions, leaving neurons vulnerable to damage. Senescent microglia are also more reactive to stimuli, referred to as microglial priming. Primed microglia exhibit a more sustained inflammatory response, contributing to the impairment of neuronal function. Given the role of microglia in aging and neurodegeneration, microglial-targeted therapies could serve as an additional approach to de-escalate neurodegenerative diseases.

### Effects of neuroinflammation in various neurodegenerative diseases

Neuroinflammation is a common feature of various neurodegenerative diseases and is characterized by microglial and astroglial activation and the secretion of pro-inflammatory mediators such as ROS, NO, and various pro-inflammatory cytokines and chemokines including TNF-α, IL-1β, and IL-6.^21^ Several stimuli may initiate neuroinflammation, including Pathogen-Associated Molecular Patterns (PAMPs), typically found in infected tissues, and Damage-Associated Molecular Patterns (DAMPs), such as misfolded or aggregated proteins or mislocalized nucleic acids.^22^ These stimuli are recognized by cells in the CNS, which express pattern recognition receptors (PRRs), not only mainly comprising microglia but also including perivascular and peripheral macrophages and other glial cells and neurons to a lesser extent.^21,23,24^ These PRRs include various TLRs and scavenger receptors, which can also form receptor complexes in the form of heterodimers or trimers, initiating a pro-inflammatory signaling cascade that leads to microglial activation and recruitment and generates production of neurotoxic molecules that contribute to neurodegeneration.^25–28^ When there is a persistent presence of DAMPs, as in neurodegenerative diseases, the neuroinflammatory response becomes chronic, leading to morphological, phenotypical, and functional changes in microglia and astrocytes, and the sustained release of pro-inflammatory mediators that exacerbate neurodegeneration.^23^ This chronic neuroinflammation is a critical part of almost all neurodegenerative diseases. Interfering with the initial binding of DAMPs to PRRs is a potential therapeutic strategy for various neurodegenerative diseases. Acute neuroinflammation can be beneficial in certain circumstances, leading to the stimulation of myelin repair, removal of toxic aggregated proteins and cell debris, or the secretion of protective and reparative neurotrophic factors as a causative or exacerbating factor or as a secondary component.^23,29^

AD is a progressive neurodegenerative disorder and the most common cause of dementia in older adults.^30^ The hallmark pathologies of AD are the accumulation of intracellular neurofibrillary tangles of the protein tau and extracellular plaque deposits of the Aβ peptide, the deposition of which could be attributed to the dysfunction of certain transporter molecules such as P-glycoprotein or the changes in expression levels of relevant receptor proteins such as TLRs.^31,32^ Fibrillar forms of Aβ found in these plaques have neuroinflammatory effects, triggering the accumulation and activation of microglia that surround the plaques.^26,33^ Microglia play an important role in Aβ clearance, by endocytosing and degrading both soluble and fibrillar Aβ.^34^ The chronic accumulation of Aβ and its interaction with microglial receptors such as scavenger receptor A1, CD36, CD47, CD14, and various TLRs activate microglia and initiate the aforementioned pro-inflammatory signaling cascade.^28,33^ Dysregulated Aβ clearance has been identified as a major pathway in the pathology of sporadic AD cases, particularly in aged microglia that are more prone to impaired lysosomal function.^35,36^ In later stages of AD, the pro-inflammatory cytokines produced by activated microglia downregulate genes involved in Aβ clearance, thereby enabling Aβ accumulation.^37^ Microglia can also degrade and clear the aggregated tau present in AD and other tauopathies such as progressive supranuclear palsy, frontotemporal dementia (FTD), and chronic traumatic encephalopathy (CTE).^38^ Activation of pro-inflammatory microglia contributes to the progression of tau pathology and increases tau phosphorylation, which is associated with synaptic dysfunction and cell death.^39,40^

Another major class of neurodegenerative diseases is known as synucleinopathies, which are characterized by the abnormal accumulation of the protein ASYN. These diseases include PD, the associated Parkinson's disease dementia (PDD), and dementia with Lewy bodies (DLBs).^41^ A common pathologic feature of these diseases is the onset of neuroinflammation in the areas corresponding to ASYN deposition and neurodegeneration, particularly within the substantia nigra and striatum.^42–44^ Aβ deposition has also been associated with PDD and DLP, with its presence accelerating the dementia process.^45^ In PD, which is characterized by the loss of dopaminergic (DA) neurons in the substantia nigra, extracellular accumulation and aggregation of ASYN have been shown to cause microglial activation, triggering the release of inflammatory cytokines and other neurotoxic molecules, which cause additional neurodegeneration.^46–48^ Sustained microglial activation is believed to play a prominent role in exacerbating DA neuronal loss, as the substantia nigra contains 4.5 fold larger microglial population than other brain regions, and DA neurons have reduced antioxidant capacity, rendering them susceptible to oxidative stress to a greater degree relative to other cell types within the brain.^49,50^ Chronic activation of microglia also slows the degradation of ASYN and increases its intracellular accumulation, suggesting that activated microglia are less efficient at clearing ASYN and are a critical trigger to exacerbating ASYN pathology and neurodegeneration.^51^ Cell debris from dead neurons can further attract and activate microglia, resulting in a self-perpetuating cycle of inflammation and neurotoxicity.

ALS is a progressive neurodegenerative disease characterized by the loss of motor neurons.^52^ A common hallmark of the disease shared by sporadic and familial ALS patients is the strong activation and proliferation of microglia found at sites of motor neuron loss.^53^ Microglial activation may be triggered by the accumulation of aggregates of mutant superoxide dismutase (SOD1).^22^ These activated microglia demonstrate neuroprotective properties during the early pre-symptomatic stages of ALS but shift to a more pro-inflammatory phenotype as the disease progresses, promoting neurodegeneration.^54,55^

Targeting microglial activation states by suppressing pro-inflammatory neurotoxic effects of the classically activated phenotype and/or simultaneously enhancing the anti-inflammatory, neuroprotective functions of the alternately activated phenotype are gaining promise as a therapeutic approach for neurodegenerative diseases.^56^ A rational approach to inhibiting microglial activation induced by the accumulation of disordered proteins would rely on interrupting the key steps via which these proteins interact with and activate microglia, while, at the same time, maintaining protein clearance by microglia through inhibition of aggregation in extracellular spaces.

## NANOTHERAPEUTICS IN THE CNS

### CNS drug delivery crossing the blood-brain barrier

In order for a therapeutic agent to be effective, it needs to reach the site of pathology, i.e., the CNS, where the degeneration of neurons and neuroinflammation take place. The discovery of treatments for CNS disease has been challenged by the existence of the blood-brain barrier (BBB), which is a highly selective barrier that isolates the CNS from systemic circulation, protecting the brain from pathogens and maintaining CNS homeostasis to allow proper neuronal function. The BBB is mainly made up of brain capillary endothelial cells (BCECs), which are connected to each other by tight junctions (TJs) and adherens junctions (AJs).^57^ Lacking fenestration, these BCECs are tightly packed, restricting the paracellular diffusion of hydrophilic small molecules.^58^ The transport of necessary nutrients and certain drugs across the BBB is regulated by a series of specific transport mechanisms, which can generally be classified into the following categories: passive diffusion, carrier-mediated transport, and vesicular trafficking, such as receptor-mediated transcytosis and adsorptive-mediated transcytosis.^59^ Small molecule drugs make up a large majority of available CNS therapeutics, most of which penetrate the BBB via passive diffusion, while only a small number penetrate via carrier-mediated mechanisms.^60^ One of the challenges in designing BBB-crossing small molecules is to maintain molecules' high lipid solubility while enabling reasonable solubility in aqueous brain interstitial fluid to reach target cells. This property requirement screens out more than 98% of all US Food and Drug Administration (FDA)-approved small molecule drugs to be used in the CNS.^59,60^ Compared to small molecule therapeutics, biologic drugs, such as recombinant proteins, antibodies, or nucleic acid drugs, are larger in size and generally do not cross the BBB via passive diffusion.^61^ Proper delivery vehicles needs to be tailored to carry biologic and small molecule drugs that cannot penetrate the BBB, facilitating their entry into the CNS via receptor-mediated transcytosis.^62^

Without proper drug delivery platforms to facilitate BBB crossing, CNS drugs may be delivered via alternative routes such as cerebrospinal fluid (CSF) injection and intra-cerebroventricular delivery to bypass the BBB.^61^ However, drugs delivered via these administration routes have rarely obtained FDA approval and concerns have been raised regarding the limited drug penetration into brain parenchyma from the CSF, which results in the exponential drug concentration decrease in the CNS following injection.^63^ To date, the majority of CNS-targeted pharmacological interventions have focused on administering therapeutics systemically via intravenous injection or oral administration due to the relative non-invasiveness of these delivery methods compared to local administration.^64–67^ For systemic drug delivery, the therapeutic efficacy of a drug is strongly associated with the time window in which the drug concentration is maintained above its therapeutic level without systemic toxicity.^68^ The design of a controlled drug delivery system offers an alternative strategy to maximize drug action with minimized toxicity utilizing existing therapeutic molecules.

### Additional challenges in CNS drug delivery

In addition to overcoming the BBB, other two aspects challenging the discovery of CNS therapeutics are^1^ maintaining stability of drugs in their active form before reaching the site of pathology and^2^ designing molecules targeted toward surface receptors and associated pathways of interest to reduce off-target effects.^69^ While either aspect could be addressed via structural modifications of the drugs, such as designing prodrugs that can be metabolized into a pharmacologically active drug after administration or drugs that target certain domains on receptors of interest, overcoming both obstacles presents major challenges in CNS drug design.^70,71^

Considering the difficulties in tackling all the challenges mentioned above with single therapeutic agent molecules for CNS drug delivery, it is critical to engineer drug delivery systems that could be tailored to facilitate BBB crossing of therapeutics, control the sustained release of active agents at the site of pathology, and maintain the chemical and physical stabilities of the drug. These criteria for designing drug delivery systems can all be fulfilled with nanoparticle formulations, which are designed to deliver therapeutics to the site of pathology in a targeted manner, while maintaining the unmodified structure of the active agent in an extended time-window with minimized toxicity and side effects.^72^ Nanoparticle-based drug delivery systems present great potential and offer a unique solution to the challenge of BBB penetration owing to their flexibly manipulated physical and chemical properties. Nanoparticles can be engineered to overcome the challenges that small molecule drugs face through surface functionalization with BBB-targeting transporters, loading of drugs that either cannot cross the BBB or lack structural stability and enabling controlled release at the site of pathology.^73,74^

### Overcoming the CNS drug delivery challenges with nanoparticles

Nanoparticles (NPs) refer to particles for which one or more external dimensions are in the size range of 1–100 nm for at least 50% of the particles according to the European Commission's Recommendation.^74,75^ Compared to bulk materials, nanoparticles' high surface area to volume ratio enables not only increased cellular interaction and reactivity but also concentrated loading of large amounts of therapeutic agents with minimized toxicity. Key advantages of using NP formulations for CNS delivery include an extended half-life, enhanced deposition of drug within a targeted region, and reduced side effects.^76,77^ The functionality of NPs can be tailored by modifying various characteristics such as charge, size, and surface chemistry, in addition to encapsulation of a desired drug payload.

Based on their material composition, NPs can be roughly categorized into two classes: organic (including polymeric NPs and liposomes) and inorganic (including metal NPs and carbon-based NPs).^72^ In addition to NP formulations' usage as drug delivery vehicles, the nano-scale packing of material also imposes unique physical properties compared to bulk material. One example is NanoTherm^®^, an iron oxide NP-based therapeutic for intratumoral thermotherapy in glioblastoma patients, which has superparamagnetic properties that are used for local heat generation in combination with chemotherapy to prevent tumor growth.^78^ More recent research investigations have illustrated the use of cell-derived NPs, specifically the engineering and re-engineering of exosomes, a group of extracellular vesicles, as potential drug delivery platforms.^79^ Exosomes offer unique characteristics including low immunogenicity, biodegradability, and the ability to cross many biological barriers.^80^

NPs can be engineered to target BBB transport mechanisms mentioned before in order to efficiently cross the BBB, while carrying a therapeutic drug payload. The physicochemical properties of these NPs determine the specific mode of transport across the BBB. While the vast majority of NPs are unable to cross the BBB without functionalization, there are some exceptions such as gold NPs, which have been shown to cross the BBB via passive diffusion through ion channels, and silver and titanium dioxide NPs, which can travel into the brain by decreasing transendothelial electrical resistance and disrupting the tight junctions between BCECs.^81,82^ Crossing the BBB in this manner is size dependent, with NPs being less than 10 nm in diameter. There are also various methods of temporarily disrupting the permeability of the BBB to enable NP delivery, such as through the administration of ultrasound energy or hyperosmotic agents.^83,84^ Although these methods can improve the delivery of various therapeutics into the brain, these temporary disruptions in BBB integrity could allow the passage of toxic substances into the brain, which could affect the normal functions of the CNS.

Several types of cationic NPs have been reported to interact with the negatively charged surface of the BCECs and cross the BBB via adsorptive-mediated transcytosis. There are different methods of conferring a positive charge on the surface of NPs. One such method is by fabricating NPs from multiple components that have a positive charge at physiological pH. Single component NPs have also been synthesized using cationic polymers such as chitosan or polyethylenimine (PEI) and successfully used for brain delivery.^85,86^ Positively charged polymers are well suited for delivery of negatively charged nucleic acids since NPs can easily be assembled using these components via polyelectrolyte complex formation or controlled coacervation.^87,88^ The NP surface can be functionalized with positively charged molecules such as PEI or cell-penetrating cationic peptides, i.e., TAT peptides [transduction domain of human immunodeficiency virus type-1 (HIV-1)].^89,90^ Although cationic NPs can improve transport across the BBB, such NPs can have toxic effects.

NPs can be modified or conjugated to ligands that will bind to receptors on BCECs, acting as a “molecular Trojan horse” and resulting in transport across the BBB via receptor-mediated transcytosis.^91^ This approach is acknowledged as having the most likely chance of successfully crossing the BBB.^92^ Coating the surface of NPs with polysorbate 80 facilitates adsorption of apolipoprotein E (ApoE) from blood plasma, causing the coated NPs to bind to low density lipoprotein (LDL) receptors on the endothelial cells and cross the BBB via receptor-mediated transcytosis.^93,94^ ApoE itself has been covalently attached to human serum albumin NPs, promoting the rapid uptake of these NPs into the brain.^95^ Insulin and antibodies against the insulin receptor have also been used as BBB-targeting ligands for NPs.^96^ The transferrin receptor (TfR) is the most widely studied receptor for targeting the BBB for receptor-mediated transcytosis. NPs decorated with transferrin or lactoferrin, a protein within the transferrin family, have been successfully delivered to the brain after intravenous injection.^97,98^ In order to avoid competition with the abundant amount of endogenous transferrin circulating in the bloodstream, monoclonal antibodies against transferrin receptors have also been used as targeting ligands. The antibodies OX26, 8D3, and RI7217 have been used for successful TfR-targeted brain delivery in rodents.^99,100^

In order to increase transport efficiency across the BBB, it can be advantageous to target multiple transport mechanisms when designing NPs. Several dual mechanism-targeting NPs have been used for delivery across the BBB, such as magnetic NPs embedded with transferrin, which demonstrated a synergistic effect resulting in a 50%–100% increase in BBB crossing compared to NPs targeting only one transport mechanism.^101^ NPs can also be conjugated with ligands targeting multiple receptors on the BCECs, such as the example of chitosan NPs covalently conjugated to transferrin and bradykinin B2 antibodies.^102^ These NPs were amenable to uptake via adsorptive-mediated transcytosis due to the cationic chitosan and could also bind to the TfR and bradykinin B2 receptor for uptake via receptor-mediated transcytosis.

Another non-invasive NP delivery route is intranasal (IN) administration, which is a method that bypasses systemic circulation and the BBB to achieve direct brain delivery of therapeutics, leading to high CNS concentrations of therapeutics and low systemic accumulation.^103–106^ Nanotherapeutics can pass across the nasal epithelium into the brain via two different pathways. The extracellular pathway, which is the primary mechanism for brain delivery of therapeutics, involves passive transport across the nasal epithelium.^107^ The intracellular pathway involves endocytosis into the olfactory and trigeminal nerve branches and subsequent axonal transport into the brain.^107^ Although there are some limitations to this drug delivery route such as low absorption of therapeutics, high mucociliary clearance, and enzymatic degradation within the nasal cavity or during passage across the epithelial barrier, NPs can be used as delivery vehicles to protect encapsulated therapeutics, facilitating uptake and passage into the CNS.^23,108^

To improve the NPs' serum stability without compromising the drug loading capacity, the surface of NPs can be functionalized with additional polymers such as polyethylene glycol (PEG), the hydrophilic property of which can not only improve the bioavailability of the drug but also maintain therapeutic agent's stability before reaching cellular targets of interest by protecting it from serum proteins.^109^ The sustained release of loaded active agents from the nanoparticle structure can be a result of exposure to the physiological environment, in which case the dominant interaction force enabling the formation of NPs and encapsulation of active agents could be outcompeted by the change in pH, ion concentrations, or the presence of cleavage enzymes.^110,111^

## TARGETING MICROGLIA USING NANOTHERAPEUTICS

### Current therapeutic strategies for targeting microglial mediated inflammation

**FIG. 3.**
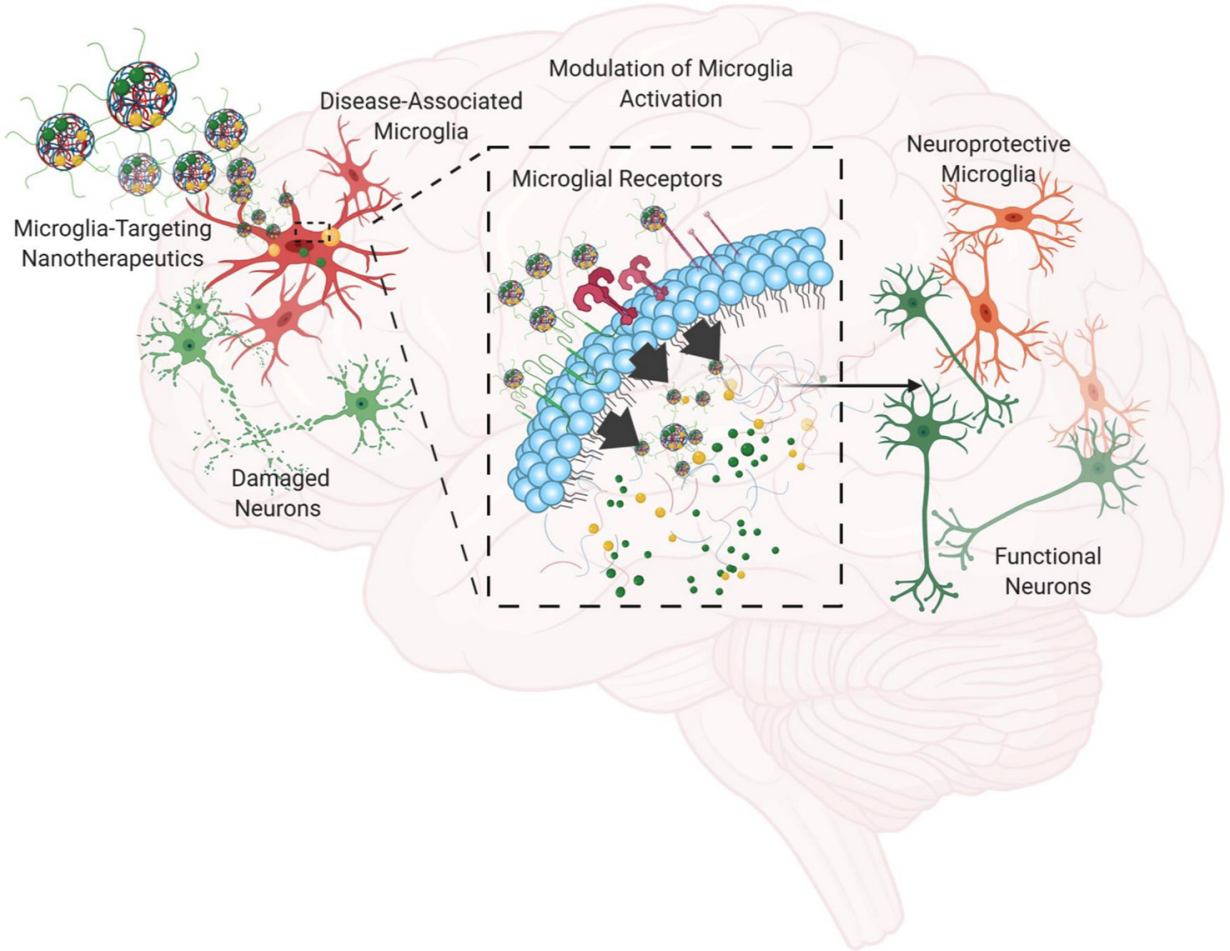
Utilizing microglia-targeting nanotherapeutics for modulation of neuroinflammation and neurodegeneration. Microglia will be chronically activated upon prolonged exposure to stimulants, and the subsequent release of neurotoxic factors from microglia will result in neuron damage. By introducing nanotherapeutics that are specifically designed to target microglia while delivering anti-inflammatory agents and protein aggregation inhibitors, microglia-associated neuroinflammation can be modulated and neurodegeneration can be slowed down.

The advances in nanomaterial-based pharmacological platforms are particularly beneficial for developing efficacious therapeutic approaches for neurodegenerative diseases, with improved cellular targeting and controlled drug release properties. Current pharmacological therapies have primarily focused on neurons as the primary targets for neurodegeneration, since the loss and dysfunction of neurons, particularly DA neurons in PD and cholinergic neurons in AD, have been found to be the primary contributor to major motor and cognitive symptoms.^66,67^ Alternatively, microglia are a promising therapeutic target, as they are an essential cell type involved heavily in protein trafficking, aggregation, and clearance and are closely associated with the neuroinflammation process.^6^ As the resident immune cell in the CNS, microglia are the first responders to changes in the tissue environment and can generate adaptive or innate immune responses upon detection of invading pathogens.^112^ From the NP therapeutic perspective, microglia can be targeted conveniently with different types of NPs due to their intrinsic phagocytic nature as immune cells. However, microglial activation has been a concern regarding the use of NPs in the CNS.^113,114^ In order to utilize nanotherapeutics to target microglia with enhanced therapeutic efficacy, more intricate design of NPs is needed for enhanced cellular targeting to deliver active agents and modulate microglial activation as a result of NP administration while minimizing off-target toxicity (Fig. 3).

Due to the involvement of microglia in neurodegeneration, potential avenues have been explored to modulate microglial pathology by either preventing early stage microglial activation or correcting pathological microglial function upon occurrence via cellular or system level intervention. Microglia participate in pathological protein processing and assist in maintaining the balance of various protein conformers and their oligomers in the intracellular and extracellular space within the CNS. To prevent toxic protein aggregate accumulation in the CNS, researchers have investigated the use of small molecules, peptides, and antibodies as inhibitors of protein aggregation, one group of which are antioxidant molecules.^115–117^ For example, epigallocatechin gallate (EGCG) belongs to a class of polyphenolics from plant extracts and has been shown to inhibit *in vitro* Aβ, tau, and ASYN aggregation.^118–121^ Its ability to prevent aggregation also translates to reducing toxic protein aggregate-induced cytotoxicity in neuroblastoma cells *in vitro*, by interfering with the membrane association of protein aggregates in order to maintain cell membrane integrity.^122^

In addition to protein aggregation, antioxidant molecules can also decrease cellular oxidative damage via active scavenging of free radicals and modulating the activity of enzymes involved in free radical production.^123,124^ Some examples are resveratrol, curcumin, and ferulic acid, all of which quench free radicals via electron transfer and protect cellular components such as DNA, RNA, and cell membranes from ROS/RNS-induced oxidative damage.^123,125^ In addition to chemical reactions, certain antioxidants such as resveratrol and EGCG have also demonstrated direct control in free radical-producing enzyme expression including NADPH oxidases, which has been shown to be responsible for microglial ROS-induced neuron death in PD.^123,126^ Besides antioxidants, non-steroidal anti-inflammatory drugs (NSAIDs), a group of compounds known for their inhibitory activity on inflammation-related enzymes including prostaglandin and cyclooxygenase, have been investigated for their ability to reduce microglial activation and protect neurodegeneration.^127^ While clinical investigation demonstrated that NSAIDs do not counteract AD, the early stage and chronic use of NSAIDs may inhibit build-up of Aβ and reduce the risk of developing PD.^128–130^ However, the use of NSAIDs after neurodegeneration takes place may accelerate disease progression as NSAIDs may interfere with microglial activity in toxic protein clearance.^128^

Specific microglial receptors have also been associated with the regulation of microglial neuroinflammation and neurodegeneration. For example, triggering receptor expressed on myeloid cells 2 (TREM2) has been shown to have increased expression in Aβ plaque-associated microglia, and the microglial response to aggregated protein can be remodeled via manipulation of TREM2 expression.^131,132^ A soluble form of TREM2 receptor protein (sTREM2) was found in the cerebrospinal fluid, and *in vivo* research in the AD mice model has shown that sTREM2 can modulate pathological microglial phenotypes.^133,134^ Modulation of microglial function by sTREM2 could be due to the competition of sTREM2 with its insoluble membrane-bound form to interfere with TREM2 involvement in protein aggregation clearance or the unknown biological function of sTREM2, which partially blocks the TREM2 signaling pathway.^133^ Another example is TLR4, whose involvement in the uptake and intracellular aggregation of Aβ and ASYN potentially initiates microglial activation during neurodegeneration, which has drawn great attention in the past decade.^135^ In vivo research in a mouse model of motor neuron degeneration has shown that chronic treatment with the TLR4 antagonist VB3323 can decrease microglial activation and improve motor function.^136^

### Mechanisms of NP uptake by microglia

Understanding the mechanisms of NP uptake by microglia can aid in the design of appropriate nanomedicines for use within the CNS. NPs interact with the microglial cell membrane and are internalized mainly through endocytosis, which is an active transport mechanism that facilitates the uptake of extracellular materials via membrane invagination.^137^ Endocytosis can be broadly divided into phagocytosis, the process by which larger particles are internalized, and pinocytosis, which can be further subdivided into the categories of clathrin-mediated endocytosis, caveolin-mediated endocytosis, clathrin- and caveolin-independent endocytosis, and micropinocytosis.^138^ In addition to these active internalization processes, NPs can also enter cells through passive diffusion. Mechanisms of NP uptake may vary depending on the activation state of microglia. Lipopolysaccharide (LPS)-activated microglia show higher *in vitro* uptake of dendrimers approximately 4–10 nm in size compared to resting microglia, which can be attributed to increased endocytosis in activated microglia.^139–141^ The physicochemical properties of NPs such as the size, surface chemistry, surface charge, and shape are important design criteria that influence the binding to and uptake by microglia, which can affect intracellular delivery of therapeutic cargo and the subsequent biological response.^142–145^ After *in vivo* administration, serum proteins adsorb on the surface of NPs, forming a layer known as the protein corona.^146^ The surface characteristics of NPs affect the identity, thickness, and orientation of the protein corona, which, in turn, also significantly affects cell interactions and uptake.^146–148^ The morphology of NPs may also affect their uptake by microglia.^142^ Independent of surface coating, spiky “urchin-shaped” gold NPs (which have numerous “bumps” and “thorns” covering the surface) showed a significantly greater extent of microglial internalization compared to spherical or rod-shaped gold NPs.^143^ Further, rod and urchin NPs caused transient microglial activation, with studies indicating that the mechanism of activation was also shape-dependent.^143^

### NP design criteria for microglial targeting

**FIG. 4.**
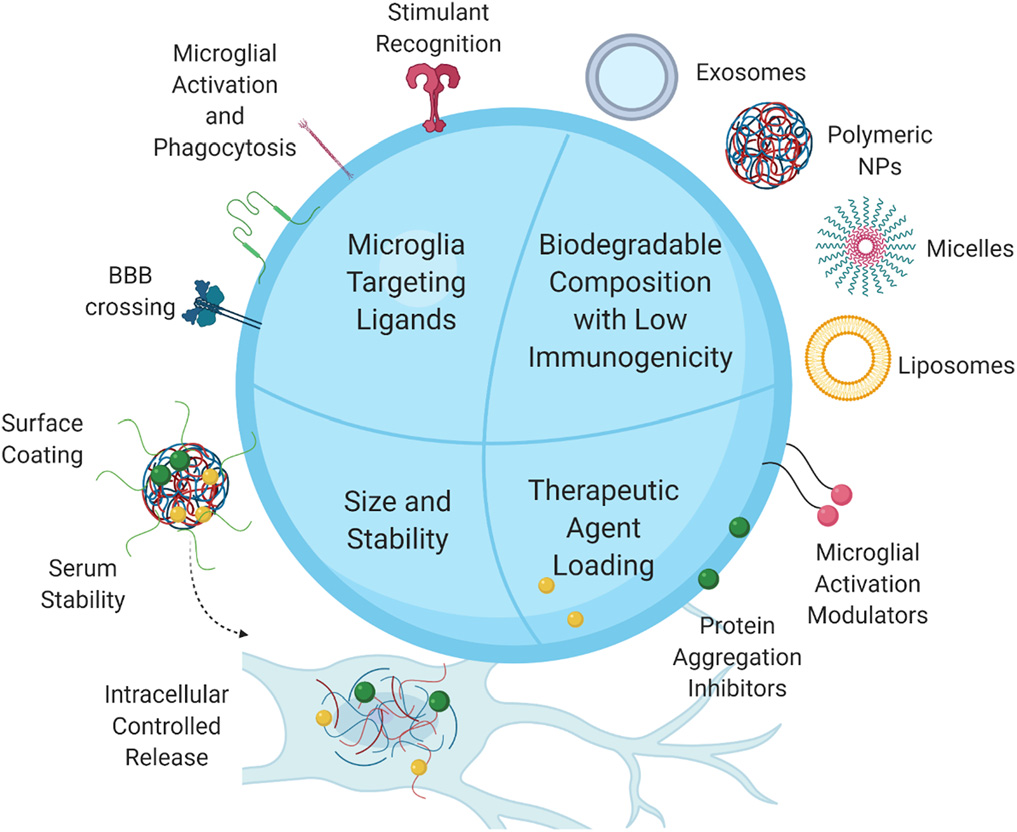
Microglia-targeting nanoparticle design criteria. To achieve modulation of neurodegeneration associated with microglia-induced neuroinflammation, nanoparticles should be designed incorporating^1^ microglial receptor-targeting ligands taking advantage of microglia's phagocytic nature,^2^ therapeutic agents for inhibition of microglial activation and toxic protein aggregation, and^3^ biodegradable materials with low immunogenicity,^4^ without compromising the nanoparticle size and serum stability.

As described above, advances in nanotechnology have enabled the design of nanotherapeutic platforms with the ability to cross or bypass the BBB, and the tunability of NPs built on these platforms ensures the delivery of therapeutics via the BBB through a variety of mechanisms.^149^ Here, we will expand the following discussion on NP design criteria unique to microglial targeting. These criteria are meant to be taken into consideration along with BBB penetration benchmarks (Fig. 4).

Microglia possess a wide range of receptors that sense exogenous or endogenous CNS disturbance and initiate a tailored immune response.^150^ By incorporating targeting ligands or peptides specific for microglial receptors involved in neurodegeneration onto or within the NP structure, the targeting capabilities of nanotherapeutics would be greatly improved and, thus, more efficiently deliver therapeutic agents with minimal side effects. PRRs expressed on microglia are a key group of receptors that not only facilitate the membrane-association and aggregation of different species of Aβ and ASYN proteins but also have elevated expression levels upon cellular disturbance.^151,152^ In addition to the TLR receptors mentioned previously, receptors for advanced glycation endproducts (RAGE) and scavenger receptors are also considered as PRRs.^151^ A previous study investigated the use of TLR4 receptor-targeting liposomes to improve the delivery of minocycline treatment in a mouse model of ALS with spinal neuron degeneration.^153^ The surface of the liposomes was modified with LPS, a TLR4 agonist, to facilitate delivery to microglia. Results showed that this targeting ligand significantly increased the uptake of encapsulated drug compared to non-targeted liposomes and that the microglia-targeted liposomes significantly delayed disease progression.^153^ Scavenger receptors, such as SR-A1 and CD36, are also PRRs that are involved in modulating aggregated protein interaction with microglia and subsequent activation. Previous research has demonstrated the uptake of different NPs through scavenger receptors, such as polystyrene-based NPs and silver NPs.^154^ NPs can also be designed as scavenger receptor ligand mimetics, such as lipoproteins and long chain fatty acids to achieve specific targeting.^154,155^ Other microglial receptors have also been investigated as potential avenues to deliver nanotherapeutics to microglia specifically. For example, Lee *et al.* studied the use of ceria-zirconia NPs (CZ NPs) for inhibiting microglial activation in a neuropathic pain mouse model.^156^ The NP surface was decorated with CD11b antibody via NHS-ester conjugation, and *in vitro* results showed that these NPs had much higher microglial internalization than NPs with isotype control antibody conjugates.^156^ Brain slides obtained after intrathecal administration of CZ NPs into mice showed that microglia had significantly higher uptake of NPs with antibody conjugates than any other CNS cell types.^156^

In addition to its first-responder role during the CNS inflammatory development, microglia are also highly functional as phagocytes.^157^ Previous research has shown that expression of phagocytic receptors on microglia is elevated under neuroinflammatory conditions and this microglial property is particularly beneficial for targeted delivery of nanotherapeutics to microglia.^157,158^ While the studies on NP interactions specifically with microglia are limited, a few studies have highlighted the selective uptake of NPs by microglia and macrophages considering their origin and functional similarities.^159–161^ Veglianese *et al.* demonstrated the use of poly-ε-caprolactone and PEG-based NPs (PCL-based NPs) for targeted delivery of minocycline to reduce microglia/macrophage activation.^160^ *In vitro* results showed that only activated microglia or microglia with an ameboid shape were able to internalize a significant amount of these PCL-based NPs, while resting microglia did not.^160^ Most importantly, local injections of NPs in mice with spinal cord injury (SCI) were able to shift the microglial population at the lesion site from phagocytic to a more arborized resting phenotype, suggesting a reduction in the overall level of microglial activation.^160^ Similarly, another study conducted in the following year explored the use of an alternative polymeric NP formulation composed of polymethylmethacrylate (PMMA) for the treatment of SCI and both studies took the microglia/macrophage activation and its heightened phagocytic ability involved in the disease development as an opportunity for targeted drug delivery.^160,161^

NPs can also be designed to incorporate inhibitor molecules for enzymes involved in microglial inflammatory pathways during the neuroinflammatory response in PD and AD.^162^ For example, DNL747, a small-molecular inhibitor of receptor-interacting serine/threonine-protein kinase 1 (RIPK1)—a kinase enzyme involved in the downstream signaling of the TNF-α receptor, is currently under phase I clinical trial investigation for its efficacy in modulating microglial activation and related neuroinflammation in patients with AD.^163^ Preliminary research has demonstrated that RIPK1 mediates the occurrence of disease-associated microglial phenotypes, reduction in microglial phagocytic activity, and the etiology of AD.^164^ Anti-aggregation small molecules could also be incorporated within the NP formulation to improve the bioavailability of hydrophobic molecules and further valorize the NP as a drug delivery vehicle. Zheng *et al.* have compared the Aβ aggregation and ROS-inhibiting properties of resveratrol and selenium NPs functionalized with resveratrol.^165^ *In vitro* evaluation showed that functionalization with resveratrol significantly strengthened the inhibition of copper ion-induced Aβ aggregation, ROS production, and toxic protein aggregates associated with neuron death.^165^ More recently, Ren *et al.* have demonstrated the use of a mitochondria-targeted polymeric material-coated quantum dot nanoparticle to scavenge free radicals released from microglia induced by Aβ aggregation, modulating microglial activation and ameliorating neuron death *in vivo.*^166^ The incorporation of lipophilic (3-carboxypropyl)triphenyl-phosphonium bromide (TPP) molecule within the polymer shell not only enabled the mitochondria targeting ability of NPs but also strengthened the free radical scavenging ability of these NPs.^166^

In addition, NP design should also ensure reasonable colloidal stability of the NP structure and encapsulated agents in serum, which will ensure their activity once it has reached the target site and can potentially reduce side effects compared to untargeted therapeutics. This is particularly challenging for NPs decorated with targeting ligands on the surface since non-specific interactions will likely occur between targeting ligands and other proteins or receptors in serum or other cells in the CNS and modify its structure before reaching the site of pathology. One of the modifications made to improve NP stability is with PEG, a long chain hydrophilic polyether compound, which improves the NP's structural and chemical stability and performance in physiological environments.^167,168^ The PEG shields the active agent within the NP core from enzymatic degradation and reduces nonspecific protein binding of NPs, and the hydrophilic nature of PEG increases the circulation time of the therapeutic.^167,169^ With these criteria taken into account, NPs could be designed to have high bioavailability to microglia and improved pharmacodynamic profiles when used via systemic administration.

### Addressing NP-mediated toxicity

The unique physicochemical properties of NPs, which can be markedly different from bulk materials, can lead to unpredictable interactions with cells and tissues and subsequent neurotoxic effects.^170,171^ Neurotoxicity can be induced through direct effects on the morphology or function of cells in the CNS or by triggering glial activation and affecting the interactions between microglia and neurons, leading to neurological damage and cognitive or behavioral impairments.^170,172^ Most of these effects are due to oxidative stress, with other mechanisms of neurotoxicity including inflammation, apoptosis, alterations in gene expression and signaling pathways, and epigenetic modifications.^173^ Various physicochemical properties of NPs can contribute to toxic effects, including the size, shape, chemical composition, surface chemistry, and aggregation. Cationic NPs have been widely shown to have toxic properties, through binding to serum proteins and disrupting the structure and function of cell membranes, including forming holes and eroding/thinning the membrane.^174^ Many commonly used metallic NPs, such as gold, silver, silica, iron oxide, and titanium dioxide, have been reported to have neurotoxic effects.^175^ Some studies have found that pre-treatment or co-treatment with antioxidants can inhibit the inflammatory response to metallic NPs, reducing apoptosis and protecting against NP-induced neurotoxicity.^175–177^ Antioxidants can also be incorporated within NPs for controlled release. Polymeric NPs have several advantages including controlled release, biodegradability, specific cell targeting, and the ability to protect encapsulated drugs, which makes them attractive for use as drug delivery vehicles to the CNS.^178,179^ Their disadvantages include aggregation and potential neurotoxicity, which could be caused by the degradation process and residual byproducts.^178^

Strategies to attenuate neurotoxicity include controlling the NP size and shape, coating the NPs to modify the surface chemistry, and removing toxic components from the fabrication process.^178^ Surface coating with biocompatible polymers, such as PEG, can create a protective hydrophilic layer around the NPs, shielding positive surface charges, which will reduce toxicity, and also extending the circulation time of systemically administered NPs by delaying clearance via the reticuloendothelial system.^180,181^ Adding targeting ligands on the surface of NPs is another approach to reduce toxicity since targeted NPs have improved efficacy at much lower concentrations compared to untargeted NPs.^180,182,183^ Targeting ligands specific for microglia will also improve the binding and uptake into these cells, enhancing the desired biological response. Extensive characterization and *in vitro* evaluation can and should be conducted prior to selecting a particular NP formulation for use *in vivo* in order to minimize or avoid potential neurotoxicity.

There are various methods to assess nanotoxicity *in vitro*, including evaluation of the effects on cell viability and proliferation, apoptosis and necrosis, oxidative stress, and genotoxicity.^184^ NP toxicity can be assessed using various neural cell culture models in order to determine the potential adverse effects of NPs in the brain, including monolayer cell cultures, co-cultures, and tissue slices. Cultures including primary brain cells are more likely to accurately recapitulate the morphology and function of the respective cells *in vivo* compared to immortalized cell lines.^185^ NPs can also induce toxicity outside of the brain, particularly when they are administered systemically through intravenous injection. In vivo NP toxicity should be assessed through evaluation of organ distribution and clearance, NP degradation, immunotoxicity, histopathology, and single dose/repeated dose toxicity measurements.^184^

### Using nanotherapeutics to modulate microglial activation

As the primary endogenous immune cell in the CNS, microglia constantly sense endogenous or exogenous stimuli and respond to cues by shifting its phenotype while clearing undesirable debris. Considering the sensitive nature of microglia, certain types of NPs may actually stimulate microglial activation rather than ameliorating it.^113^ Previous research has shown that silver NPs administered via the intranasal route can reach the CNS and cause subsequent NP size-dependent microglial activation in rats.^186^ From this perspective, biocompatible materials such as biodegradable polymeric NPs would be preferred since the structure is less likely to cause hyperactivity of the immune system, and its rapid degradation via physiological enzymes will reduce any toxicity cause by residual material accumulated in the CNS.^187^ More recently, researchers have also investigated the effect of human mesenchymal stem cell (MSC)-derived exosomes on microglia-mediated neuroinflammation. Not only did MSC-derived exosomes reduce pro-inflammatory cytokine production from activated microglia *in vitro*, but also intranasally administrated exosomes reached the CNS and reduced microglial activation in rats with perinatal brain injury.^188^

Considering the complex and multifaceted disease mechanisms of neurodegeneration, it would be beneficial to utilize combinatorial therapeutics targeting more than one aspect of pathology. One way to achieve this goal is to design nanotherapeutics that incorporate multiple therapeutic agents and targeting peptides or small molecule ligands to interfere with more than one inflammatory pathway in microglia. One example is a scavenger receptor-targeting amphiphilic macromolecule-based NP (AM NP) design, carrying ferulic acid-derived polymer as a therapeutic agent.^189^ These AM NPs were designed to have strong affinity to scavenger receptors, specifically CD36 and SR-A1 on microglia, and can weaken the receptor binding of ASYN, thus reducing ASYN aggregation-induced microglial activation. In vivo studies in a PD mouse model showed that the AM NPs can reduce ASYN deposition in the CNS as well as reduce microglial activation and recruitment, which were achieved by the combinatorial effect of the scavenger receptor-targeting AM shell and anti-inflammatory ferulic acid polymer components.^189^ This result highlights the use of NP formulations as a unique drug delivery platform, which not only supports but also strengthens the activity of the bioactive agent and enables targeted delivery.

An alternative approach to tackle the complex mechanism of neurodegeneration can be directed toward screening molecules that have multiple targeting enzymes in different pathways involved in microglial activation and also toward designing nanotherapeutics wisely to achieve simultaneous microglial targeting, toxic protein aggregate clearance, and neuroinflammation modulation. A bioinspired nanostructure, apolipoprotein E3-reconstituted high density lipoprotein (ApoE3-rHDL), is one example with a simple structural design, which not only binds to Aβ and facilitates protein clearance but also reduces microglial activation and neuron damage in an AD mouse model.^190^

While genetic manipulation of microglia in animal models of neurodegeneration has greatly improved our understanding of disease mechanisms, gene therapy approaches to modulate microglial activation for patients are still under investigation.^191^ For example, extracellular vesicles loaded with long intergenic non-coding RNA-cyclooxygenase-2 (LincRNA-Cox2) were shown to control LPS-induced microglial proliferation in mice, which is an early event in the development of neurodegeneration.^192^ However, similar genetic manipulations also need to take into consideration that activated microglia also contribute to clearing the CNS environment and protecting neuronal damage. In this case, while early stage microglial proliferation is controlled, in the long run, the silenced gene expression may interfere with microglia's normal function as immune cells and leave other CNS cells vulnerable to insults. Rather than silencing gene expression or deleting specific microglial population, nanotherapeutics should be designed to guide a shift in the spectrum of microglial phenotypes toward a more neuroprotective state via targeted delivery and controlled release of therapeutics to counteract microglial activation. With this taken under consideration, nanotherapeutics designed, either for early stages of neurodegeneration where microglial activity needs to be promoted or for late stages where microglial reactivity requires suppression, will not interfere with the microglial population's diversity and natural plasticity and have long-term effectiveness for patients.^193^

## CONCLUSION

Microglia are an essential cell type involved in the neuroinflammation process. The challenge in designing microglia-targeting nanotherapeutics for neurodegeneration is to identify microglia-targeting peptides or small molecule ligands, utilize biocompatible and biodegradable nanomaterials with low immunoreactivity, and incorporate agents to modulate microglial activation while maintaining population diversity. The identification of unique microglial markers and NP interaction with microglia will greatly guide the design of successful nanomedicine platforms that enable the targeted delivery of agents while minimizing off-target effects and system level toxicity.

## Data Availability

Data sharing is not applicable to this article as no new data were created or analyzed in this study.
